# Epithelial cell senescence impairs repair process and exacerbates inflammation after airway injury

**DOI:** 10.1186/1465-9921-12-78

**Published:** 2011-06-10

**Authors:** Fang Zhou, Shigemitsu Onizawa, Atsushi Nagai, Kazutetsu Aoshiba

**Affiliations:** 1 Pulmonary Division, Graduate School of Medical Science, Tokyo Women's Medical University, 8-1 Kawada-cho, Shinjuku-ku, Tokyo 162-8666, Japan; 2 First Department of Medicine, Tokyo Women's Medical University, 8-1 Kawada-cho, Shinjuku-ku, Tokyo 162-8666, Japan

## Abstract

**Background:**

Genotoxic stress, such as by exposure to bromodeoxyuridine (BrdU) and cigarette smoke, induces premature cell senescence. Recent evidence indicates that cellular senescence of various types of cells is accelerated in COPD patients. However, whether the senescence of airway epithelial cells contributes to the development of airway diseases is unknown. The present study was designed to test the hypothesis that premature senescence of airway epithelial cells (Clara cells) impairs repair processes and exacerbates inflammation after airway injury.

**Methods:**

C57/BL6J mice were injected with the Clara-cell-specific toxicant naphthalene (NA) on days 0, 7, and 14, and each NA injection was followed by a daily dose of BrdU on each of the following 3 days, during which regenerating cells were allowed to incorporate BrdU into their DNA and to senesce. The p38 MAPK inhibitor SB202190 was injected 30 minutes before each BrdU dose. Mice were sacrificed at different times until day 28 and lungs of mice were obtained to investigate whether Clara cell senescence impairs airway epithelial regeneration and exacerbates airway inflammation. NCI-H441 cells were induced to senesce by exposure to BrdU or the telomerase inhibitor MST-312. Human lung tissue samples were obtained from COPD patients, asymptomatic smokers, and nonsmokers to investigate whether Clara cell senescence is accelerated in the airways of COPD patients, and if so, whether it is accompanied by p38 MAPK activation.

**Results:**

BrdU did not alter the intensity of the airway epithelial injury or inflammation after a single NA exposure. However, after repeated NA exposure, BrdU induced epithelial cell (Clara cell) senescence, as demonstrated by a DNA damage response, p21 overexpression, increased senescence-associated β-galactosidase activity, and growth arrest, which resulted in impaired epithelial regeneration. The epithelial senescence was accompanied by p38 MAPK-dependent airway inflammation. Senescent NCI-H441 cells impaired epithelial wound repair and secreted increased amounts of pro-inflammatory cytokines in a p38 MAPK-dependent manner. Clara cell senescence in COPD patients was accelerated and accompanied by p38 MAPK activation.

**Conclusions:**

Senescence of airway epithelial cells impairs repair processes and exacerbates p38 MAPK-dependent inflammation after airway injury, and it may contribute to the pathogenesis of COPD.

## Background

Aging is a risk factor for chronic obstructive pulmonary disease (COPD) [1]. Recent evidence indicates that cellular senescence of various types of cells is accelerated in COPD patients, including alveolar type II cells, endothelial cells, fibroblasts, and peripheral blood lymphocytes [2-5]. Cellular senescence is a state of essentially irreversible growth arrest that occurs either as a result of a large number of cell divisions (replicative senescence) or exposure to any of wide range of stimuli, including oncogene activation, oxidative stress, and DNA damage (premature senescence) [6,7]. Unlike apoptotic cells, senescent cells remain metabolically active and are capable of altering their microenvironment for as long as they persist [6,7]. Since senescent cells accumulate in vivo, they are presumed to contribute to the pathogenesis of age-related diseases, such as COPD and atherosclerosis, in at least two distinct ways, first inhibiting tissue repair, because they remain viable but are unable to divide and to repair tissue defects, and second, by acting as a source of chronic inflammation, because senescent cells have been shown to secrete pro-inflammatory mediators [1,6-10]. However, whether the senescence of airway epithelial cells contributes to the development of airway diseases is unknown.

Clara cells are the principal progenitors of the distal airway epithelium [11-14]. Clara cells of mice and certain other species are rich in a cytochrome P450 enzyme (CYP2F2) and therefore are sensitive to the toxic effects of naphthalene (NA), which is metabolized to a toxic intermediate by the enzyme [11-14]. Repair of the airway epithelium after NA injury is accomplished in several overlapping stages. In mice, the proliferative response peaks 1 to 2 days after NA injury and is followed by the differentiation phase, which is normally completed in 2 weeks [13].

We hypothesized that senescence of airway epithelial cells impairs repair processes and exacerbates inflammation after an airway injury. To test this hypothesis, we utilized a well-established murine model of NA-induced Clara cell depletion. To induce airway epithelial cell senescence in this model, we intraperitoneally injected mice with the brominated thymidine analog 5-bromo-2'-deoxyuridine (BrdU) after NA injury. BrdU is incorporated into DNA during the S-phase of the cell cycle, and is commonly used to identify and track proliferating cells. However, emerging evidence indicates that BrdU imposes genotoxic stress that induces premature senescence and therefore limits cell's proliferative response to growth stimuli [15-18]. In this study we demonstrated that administration of BrdU following repeated exposure to NA induced epithelial cell (Clara cell) senescence and p38 mitogen-activated protein kinase (MAPK)-dependent inflammation in the distal airway epithelium of mice. These findings suggest that airway epithelial cell senescence impairs repair processes and exacerbates inflammation after airway injury, and presumably contributes to pathological alterations in the airways of COPD patients.

## Methods

### Animal protocol

The animal protocol was reviewed and approved by the Animal Care, Use, and Ethics Committee of Tokyo Women's Medical University. Eight-week-old male C57/BL6J mice were intraperitoneally injected with NA (Kanto Chemical, Tokyo, Japan: 200 mg/kg body wt) or corn oil vehicle on day 0 alone (acute model), or on days 0, 7, and 14 (chronic model). Each NA injection was followed by intraperitoneal injection of BrdU (Sigma, St. Louis, MO: 200 mg/kg body wt) or 0.3% carboxymethycellulose, on 3 consecutive days (days 1-3, 8-10, and 15-17). This BrdU administration schedule was chosen because epithelial proliferation in mice is maximal 1 to 2 days after exposure to NA [13]. The p38 mitogen-activated protein kinase (MAPK) inhibitor SB202190 (Enzo Life Sciences, Plymouth Meeting, PA) or 0.1% DMSO was administered by intraperitoneal injection 30 minutes before each BrdU injection. Animals were killed on days 1, 2, 3, 4, 11, or 28 by injecting an overdose of pentobarbital sodium [19].

### Human lung tissue samples

The protocol of the study conformed to the Declaration of Helsinki, and approval from the Tokyo Women's Medical University Institutional Review Board was obtained. Lung tissue blocks were obtained from COPD patients (*n *= 14), asymptomatic smokers (*n *= 7), and asymptomatic nonsmokers (*n *= 8) during lung volume reduction surgery or pulmonary resection for localized lung cancer. The clinical information regarding these patients is shown in Table 1.

**Table 1:** Characteristics of the subjects

	COPD patients	Smokers	Nonsmokers
	(*n *= 14)	(*n *= 7)	(*n *= 8)
Male/females, n	12/2	7/0	2/6
Age, years	65.9 ± 2.2	60.9 ± 6.3	64.3 ± 3.8
Smoking, pack years	80.0 ± 14.1^††^	50.7 ± 6.2^†^	0 ± 0
FEV1, liters	0.91 ± 0.11**	2.35 ± 0.17	2.14 ± 0.12
FEV1/FVC, %	34.0 ± 3.4**	75.4 ± 2.9	75.0 ± 4.3
FEV1, % predicted	35.5 ± 4.0**	91.0 ± 6.4	101.2 ± 5.4

The COPD patients and smokers were ex-smokers. **P < 0.01 compared to asymptomatic smokers and nonsmokers. †P < 0.05 and ††P < 0.01 compared to asymptomatic nonsmokers.

### Tissue preparation

Lungs of mice were inflation fixed in situ for 5 minutes with 10% neutral buffered formalin (NBF) at 25 cm water pressure, removed, and immersion fixed in NBF for 24 hours. Formalin-fixed tissue was embedded in paraffin, and sectioned (3 μm). For frozen fixation, lungs were inflated by manual instillation of 50% optimal cutting temperature compound, quickly frozen, and sectioned (3 μm). The tissue blocks from human lungs were fixed in NBF, embedded in paraffin, and sectioned (3 μm).

### Cell culture

NCI-H441 cells (the American Type Culture Collection, Rockville, MD), a Clara-cell-like human lung adenocarcinoma cell line, were cultured in RPMI 1640 supplemented with 10% FCS. Cells were exposed to BrdU by culturing for 10 days in the presence of BrdU (25, 50, or 100 μM), with a medium exchange on day 5; control cells were similarly cultured in the absence of BrdU. In some experiments, the p38 MAPK inhibitor SB202190 was added to a concentration 10 μM [19]. For telomerase inhibition, cells were cultured for 28 days in the presence of MST-312 (2.5 μM: Calbiochem, Gibbstown, NJ), with passages every 7 days; control cells were similarly cultured in the absence of MST-312 [20]. Cell numbers were counted manually or by Alamar^®^blue assay (Invitrogen, Camarillo, CA). Population doubling (PD) at each passage was calculated by using the formula: PD = ln (number of cells recovered/number of cells inoculated)/ln2.

### Epithelial repair assay

NCI-H441 cells were cultured on 30 mm-plates in RPMI 1640 supplemented with 10% FCS in the presence or absence of 25 μM BrdU for 10 days. Cell monolayers were then damaged mechanically by crossing three times with a 10-200 μl volume universal pipette tip (Corning, NY, USA) and epithelial repair after mechanical damage was monitored for 72 hours. (*See *Additional file 1 for details.)

### Enzyme-linked immunosorbent assay (ELISA)

The concentrations of cytokines/chemokines in the cell culture supernatants were measured by using ELISA kits (Biosource International, Camarillo, CA), and values were normalized to the number of cells.

### Senescence-associated β-galactosidase (SA β-gal) staining

SA β-gal staining was performed as described previously [21]. (*See *Additional file 1 for details.)

### Immunohistochemistry and immunofluorescence

The primary antibodies against Clara cell 10-kDa secretory protein (CC10), β-tubulin IV, Ki-67, BrdU, p16^INK4a ^(p16), p21^WAF1/CIP1 ^(p21), phospho(Thr180/Tyr182)-p38 MAPK, polyclonal anti-phospho(Ser/Thr)-ataxia teleangiectasia mutated kinase (ATM)/ataxia teleangiectasia and Rad3-related kinase (ATR) substrate, phospho(Ser139)-H2AX (γH2AX), CD45, and CD90.2 were used. For immunohistochemistry and immunocytochemistry, the primary antibodies were detected with a secondary antibody conjugated with a horseradish-peroxidase (HRP)-labeled polymer (Envison+^®^, DAKO Japan, Tokyo, Japan; Histofine^® ^Simple Stain, Nichirei Biosciences, Tokyo Japan). Immunoreactants were detected with a diaminobenzidine substrate or a HistoGreen^® ^substrate (AbCys, Paris, France). (*See *Additional file 1 for details.) For immunofluorescence staining, the primary antibodies were reacted with secondary anti-IgG antibodies conjugated with Alexa Fluor 350, Alexa Fluor 488, or Alexa Fluor 594 (Invitrogen, Carlsbad, CA). Images were acquired by using an Olympus BX60 microscope (Olympus Optical Co., Ltd., Tokyo, Japan) equipped with a digital camera, and processed with a computerized color image analysis software system (Win Roof Version 3.5; Mitani Corporation, Fukui, Japan) and Adobe Photoshop software (San Jose, CA). The numbers of γH2AX-foci in the cell nuclei of at least 50 cells were counted visually through an Olympus BX60 microscope equipped with a 100× objective as described previously [22,23].

### Immunoblot analysis

Cell lysates were fractionated by sodium dodecyl sulfate-polyacrylamide gel electrophoresis and transferred to a polyvinylidene difluoride membrane. The membrane was probed with primary antibodies against phospho(Thr180/Tyr182)-p38 MAPK, p38 MAPK, NF-κB p65, phospho-NF-κB p65 (Ser536), phospho(Ser139)-H2AX (γH2AX, Cell Signaling), p21, or actin (*See *Additional file 1 for details.)

### Cell cycle analysis

The DNA content of cells was analyzed by flow cytometry [24].

### Morphometric analysis in murine distal airways

Morphometric analysis was performed in the distal bronchiolar airway region. Since cell type representation varies with anatomical location, the analysis was limited to the final 200-μm basement membrane (BM) that ended in a well-defined bronchoalveolar duct junction [25]. The distal bronchiolar airway epithelium was defined as the cells located between the basal lamina and the airway lumen, and the peribronchiolar interstitium was defined as the cells located between the basal lamina of the distal bronchiolar airway epithelium and an adjacent blood vessel, alveolus, or bronchiole. Ten distal bronchiolar airways were randomly selected on each slide and examined under a microscope at ×400 magnification.

Epithelial injury was quantified on hematoxylin-eosin-stained slides by counting the number of necrotic bronchial epithelial cells that had exfoliated into the airway lumen and dividing the number by the total length of the BM. Clara cells were identified by immunohistochemistry for CC10, and the number of CC10-positive cells in the epithelium was divided by the total length of the BM. Epithelial cell proliferation was quantified by dividing the number of Ki-67-labeled nuclei in the CC10-positive cells by the total number of CC10-positive cells, or the number of Ki-67-labeled nuclei in the CC10-negative epithelial cells by the total number of CC10-negative epithelial cells. Epithelial cell senescence was quantified by counting the number of p21-labeled nuclei in CC10-positive cells or the number of SA β-gal-positive cells that co-express CC10 and dividing the number by the total number of CC10-positive cells. DNA damage response was quantified by dividing the number of phospho-ATM/ATR substrate-labeled nuclei in the CC10-positive cells by the total number of CC10-positive cells, or by counting the number of γH2AX foci in CC10-positive cells. Activation of p38 MAPK was quantified by dividing the number of phospho-p38 MAPK-labeled nuclei in the CC10-positive cells by the total number of CC10-positive cells. Airway inflammation was evaluated by counting the number of CD45-positive cells (pan-leukocytes) and the number of CD90.2-positive cells (T-cells) in the peribronchiolar interstitium and dividing their numbers by the total length of the BM.

### Morphometric analysis of human bronchiolar airways

Human lung tissue sections were triple immunofluorescence stained for CC10, p16, and phospho-p38 MAPK, and five microscopic fields of tissue from each patient containing a region of distal bronchiolar airway epithelium were examined under an epifluorescence microscope at ×400 magnification. The number of CC10-positive cells that stained positive for p16 was divided by the total number of CC10-positive cells, the number of CC10-positive cells that stained positive for phospho-p38 MAPK was divided by the total number of CC10-positive cells, and the number of CC10-positive cells that stained positive for both phospho-p38 MAPK and p16 was divided by the total number of CC10-positive cells. The number of CC10-positive cells that stained positive for both phospho-p38 MAPK and p16 was divided by the total number of CC10-positive cells that stained positive for p16 (p38 MAPK index for senescent Clara cells), and the number of CC10-positive cells that were positive for phospho-p38 MAPK but negative for p16 was divided by the total number of CC10-positive cells that were negative for p16 (p38 MAPK index for presenescent Clara cells).

### Statistical analysis

Data are expressed as means ± SEM. Statistical analyses were performed by using the Excel X software program with the add-in software Statcel 2 (OMS, Tokyo, Japan). Data obtained from two groups were compared by using Student's *t*-test. Comparisons among three or more groups were made by analysis of variance (ANOVA), and any significant differences were further examined by the Tukey-Kramer comparisons post hoc test. Data were tested for correlations by the Spearman rank correlation test. A p value of < 0.05 was considered significant.

## Results

### BrdU does not affect acute epithelial damage, repair, or inflammation after a single exposure to NA

**Figure 1:**
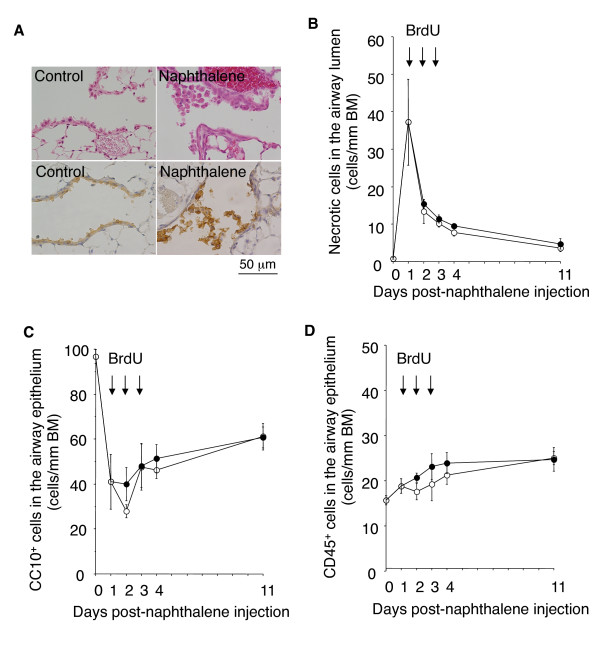
**BrdU does not affect the intensity of acute airway epithelial damage, recovery, or inflammation after a single exposure to NA**. Mice were intraperitoneally injected with NA or corn oil vehicle (day 0) and then intraperitoneally injected with BrdU or 0.3% carboxymethycellulose on days 1, 2, and 3. Animals were killed on days 1, 2, 3, 4, and 11. On days 1, 2, and 3 the mice were killed before the BrdU injection. (*A*) Hematoxylin-eosin stained (*upper panels*) and anti-CC10 immunostained (*lower panels*) lung tissue of mice on day 1 after exposure to NA or control vehicle. The lungs of the mice exposed to NA contain many distal airway epithelial cells (Clara cells) that are vacuolated, swollen, and have exfoliated into the airway lumen. (*B*-*D*) Time course of epithelial cell damage and airway inflammation after a single exposure to NA. *Open circles*: mice injected with NA alone. *Closed circles*: mice injected with both NA and BrdU. BrdU had not affected the degree of NA-induced epithelial cell damage, recovery (*B *and *C*), or airway inflammation (*D*) at any of the time points evaluated. Data are expressed as the means ± SEM. *N *= 4-5 at each time point for each group of mice. BM: basement membrane. No histological changes were observed in the lungs of mice injected with BrdU alone (photographs not shown).

We first investigated whether administration of BrdU would exacerbate airway epithelial damage after a single exposure to NA. Previous studies have shown that a single exposure to NA induces acute, selective injury of the Clara cells of the distal airway epithelium within 2 days. Acute NA injury is followed by epithelial cell proliferation and re-differentiation and normally resolves in two weeks [12-14]. As shown in Figure 1A, on day 1 after NA exposure the Clara cells of the distal airway epithelium were vacuolated and swollen, and many of the cells exfoliated into the airway lumen. Ciliated cells had become squamous and extended to cover the denuded BM. Administration of BrdU on days 1, 2, and 3 post-NA exposure did not affect the intensity of the epithelial cell exfoliation into the airway lumen (Figure 1B) or reduction and subsequent recovery in the number of Clara cell 10-kDa secretory protein (CC10)-positive cells (Clara cells) remaining within the airway epithelium (Figure 1C). No histological changes were observed in the lungs of mice exposed to BrdU alone.

NA-induced epithelial damage was followed by airway infiltration by neutrophils and mononuclear lymphocytes. BrdU did not alter the intensity of CD45-positive cell (pan-leukocytes) infiltration of the distal airways of mice exposed to NA (Figure 1D). Thus, BrdU did not affect the "acute" airway epithelial damage, repair, or inflammatory response after a single NA exposure.

### BrdU impairs epithelial regeneration after repeated NA exposure

The above findings indicated that BrdU does not aggravate NA-induced airway epithelial damage. However, previous studies showed that long-term exposure to BrdU imposes genotoxic stress that induces premature senescence and limits the proliferative response of cells to growth stimuli [15-18]. We therefore investigated whether BrdU administration to mice would eventually induce senescent growth arrest that impaired the epithelial regenerative response to repeated airway injury. To do so, mice were injected with NA once a week for 3 weeks (days 0, 7, and 14), and each NA injection was followed by administration of BrdU on 3 consecutive days (days 1-3, 8-10, and 15-17), during which regenerating cells were allowed to incorporate BrdU into their DNA and to senesce. The mice were sacrificed on day 28, which allowed the airway epithelium to recover for 14 days after the final exposure to NA.

**Figure 2:**
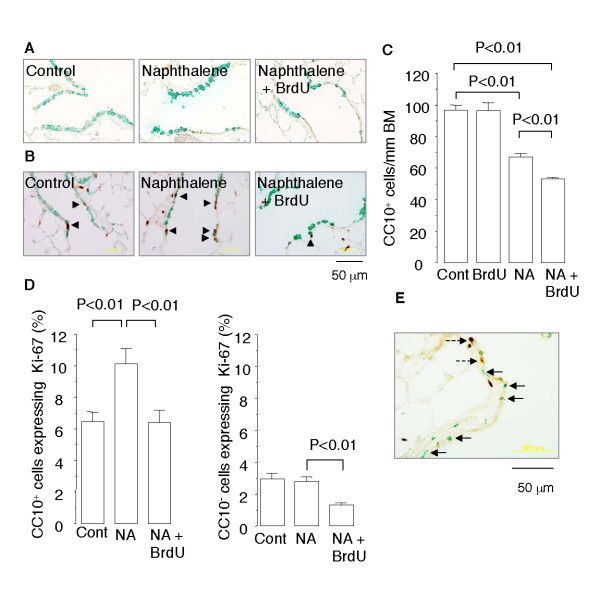
**BrdU impairs epithelial regeneration after repeated NA exposure in mice**. Mice were injected with NA once a week for 3 weeks (days 0, 7, and 14), and each NA injection was followed by administration of BrdU on 3 consecutive days. The animals were sacrificed on day 28, and lung tissue sections were double immunostained for (*A*) CC10 (*green*) and β-tubulin (*brown*), (*B*) CC10 (*green*) and Ki-67 (*brown*), and (*E*) BrdU (*green*) and Ki-67 (*brown*). *Arrowheads *indicate CC10-positive cells that express Ki-67. *Arrows *indicate cells that stained positive for BrdU. *Broken arrows *indicate cells that express Ki-67. (*C *and *D*) Quantitative analyses of the number of CC10-positive cells within the distal airway epithelium (*C*), and the proportion of CC10-positive cells that express Ki-67 and the proportion of CC10-negative cells that express Ki-67 (*D*). Data are expressed as the means ± SEM. *N *= 4-6 in each group of mice. BM: basement membrane. Panel *E *shows that very few BrdU-positive cells (*green*) stained positive for Ki-67 (*brown*).

The distal airway epithelium of the mice exposed to NA on days 0, 7, and 14 and sacrificed on day 28 was mostly composed of CC10-positive Clara cells, but occasional β-tubulin-positive ciliated cells and CC10-negative, β-tubulin-negative nondescript cells were observed (Figure 2A). The number of CC10-positive cells in the distal airway epithelium of the mice was 69% of the basal level, indicating that regeneration was still continuing when the mice were sacrificed (Figure 2C). However, in the mice exposed to NA (days 0, 7, and 14) and injected with BrdU (days 1-3, 8-10, and 15-17), the number of CC10-positive cells in the distal airway epithelium had recovered to only 55% of the basal level, indicating that regeneration was impaired.

Different cell types participate in the regenerative response to NA-induced Clara cell depletion in the distal airway, and they include surviving CC10-positive Clara cells and a subpopulation of CC10-positive epithelial cells that consists of a pollutant-resistant subpopulation of Clara cells that retain expression of CC10 (variant CC10/CCSP-expressing cells; vCE cells), bronchoalveolar stem cells (BASCs), and CC10-negative cells, such as pulmonary neuroendocrine cells (PNECs) and ciliated cells [26]. The mice that had received NA and BrdU had lower percentages of both CC10-positive epithelial cells that expressed Ki-67 and CC10-negative epithelial cells that expressed Ki-67 than the mice that received NA alone (Figure 2B and 2D). These results suggest that BrdU blunted the proliferative response of airway epithelial progenitor cells (whether CC10-positive or CC10-negative). Furthermore, 34.9% of the CC10-positive cells and 7.5% of the CC10-negative cells in the distal airway epithelium of the mice that had received both NA and BrdU stained positive for BrdU, indicating that they had divided by day 17 (the final day of BrdU administration) and incorporated BrdU into their DNA during the S-phase of the cell cycle. However, very few (< 0.1%) of the BrdU-positive cells were positive for Ki-67 (Figure 2E). Thus, the epithelial cells that had incorporated BrdU became unable to proliferate.

### BrdU induces epithelial cell senescence after repeated NA exposure

**Figure 3:**
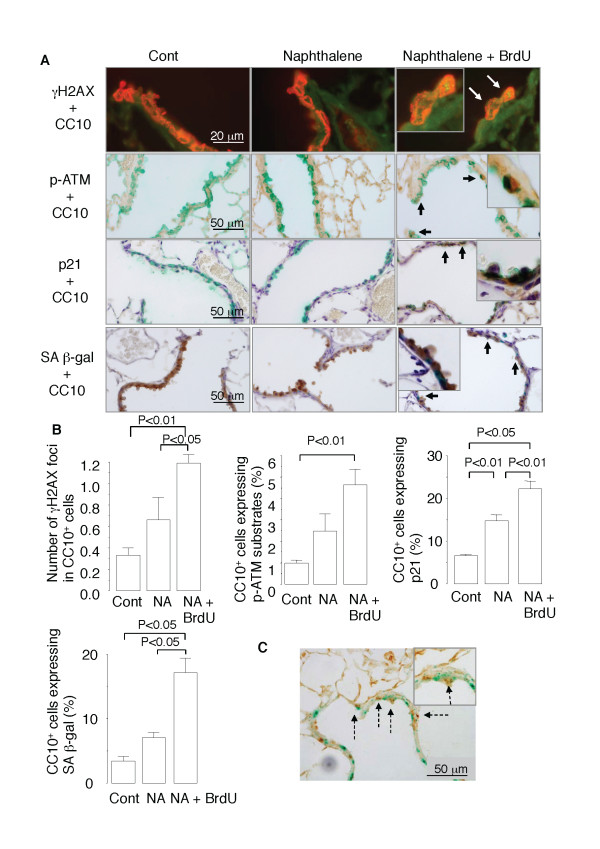
**BrdU induces epithelial cell senescence after repeated NA exposure in mice**. (*A*) Lung tissue sections were double stained for γH2AX (*green fluorescence*) and CC10 (*red fluorescence*), for phospho-ATM/ATR substrates (*brown*) and CC10 (*green*), for p21 (*brown*) and CC10 (*green*), or for SA β-gal (*green*) and CC10 (*brown*). *White arrows *indicate γH2AX foci in the nuclei of CC10-positive cells. *Black arrows *indicate CC10-positive cells that express phospho-ATM/ATR substrates, p21, or SA β-gal. (*B*) Quantitative analyses of the number of γH2AX foci in CC10-positive cells and the proportion of CC10-positive cells that express phospho-ATM/ATR substrates, p21, or SA β-gal. Data are expressed as the means ± SEM. *N *= 4-6 in each group of mice. Panel *C *shows epithelial cells that are double positive for SA β-gal (*green*) and BrdU (*brown*) (*broken arrows*)

Next, we investigated whether the impaired regeneration of the airway epithelium in the mice repeatedly exposed to NA and BrdU was attributable to induction of cellular senescence. Senescence of airway epithelial cells was detected by histological staining of lung tissue samples obtained on day 28 for different senescence markers, including phospho-ATM/ATR substrates and phospho-H2AX (γH2AX) (markers for DNA damage response), p21 (a marker for senescence growth arrest), and SA β-gal (reviewed in reference 7). γH2AX, a variant form of the H2A protein, is a component of the histone octomer in nucleosomes and phosphorylated by the kinase ATM/ATR in the phosphoinositide 3-kinase (PI3K) pathway as the first step in recruiting and localizing DNA repair proteins [22,27]. Some CC10-positive cells in the distal airway epithelium of the mice repeatedly exposed to NA stained positive for phospho-ATM/ATR, γH2AX, p21, and SA β-gal (Figure 3A), whereas 1.5 to 2 times more CC10-positive cells in the mice that had received both NA and BrdU stained positive for these senescence markers (Figure 3A and 3B). When SA β-gal-stained lung tissue samples were immunostained for BrdU, many of the SA β-gal-positive cells stained positive for BrdU (Figure 3C), suggesting that the BrdU incorporation preceded the senescence of epithelial cells. Collectively, these results suggest that BrdU induced senescence of the CC10-positive cells (i.e., Clara cells) in the airways of mice that had been exposed to NA.

### Epithelial cell senescence is accompanied by severer airway inflammation

**Figure 4:**
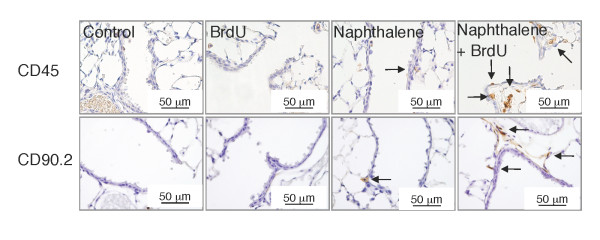
**Epithelial cell senescence after repeated NA exposure is accompanied by exacerbated airway inflammation**. Lung tissue sections were immunostained for CD45 or CD90.2 and counterstained with hematoxylin. *Arrows *indicate immunopositive cells (*brown*). Results of quantitative analyses of the numbers of CD45-positive cells and CD90.2-positive cells in the distal airways are shown in Figure 8C.

Since the repair process after NA injury is accompanied by airway inflammation, we next evaluated the severity of airway inflammation in the mice that had received NA alone or both NA and BrdU. The distal airways of the mice that had repeatedly received both NA and BrdU contained greater numbers of CD45-positive cells (pan-leukocytes) and CD90.2-positive cells (T-cells) than the distal airways of the mice that had received NA alone (Figure 4). Thus, the induction of epithelial cell senescence by BrdU was accompanied by exacerbation of airway inflammation.

### BrdU induces cellular senescence, impairs wound repair, and pro-inflammatory cytokine secretion by NCI-H441 cells

**Figure 5:**
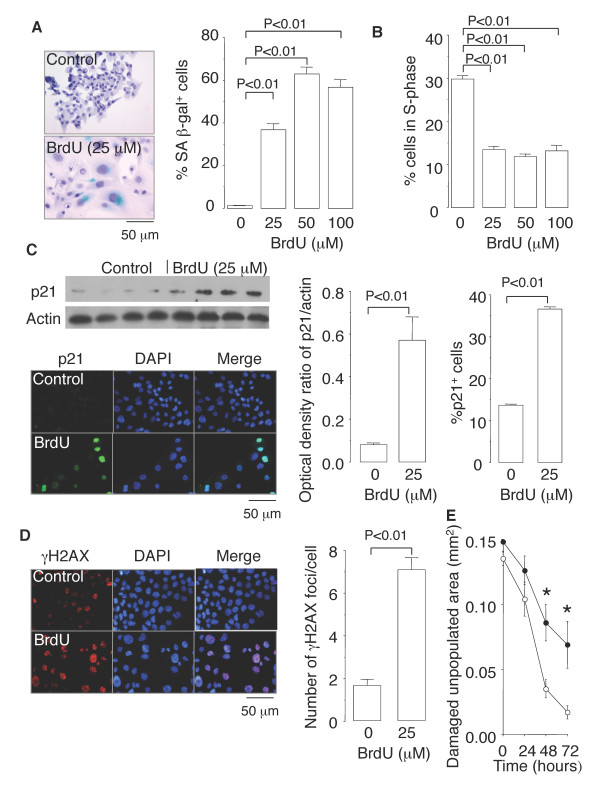
**BrdU induces cellular senescence in Clara-cell-like human lung adenocarcinoma cells**. NCI-H441 cells were exposed to BrdU at sublethal concentrations of 25, 50, or 100 μM for 10 days and evaluated for (*A*) SA β-gal activity, (*B*) cell cycle progression by flow cytometry, (*C*) p21 expression by immunoblotting and immunofluorescence, and (*D*) γH2AX expression by immunofluorescence. (*E*) Epithelial wound repair in the presence (*closed circles*) or absence (*open circles*) of 25 μM BrdU after mechanical damage of NCI-H441 cell monolayers. Data are expressed as the means ± SEM. *N *= 3-9 in each experiment. *P < 0.05 vs. cells not exposed to BrdU.

Next, we established a link that connected cellular senescence and inflammation in cultures of NCI-H441 cells, a human lung adenocarcinoma cell line with Clara cell characteristics. Trypan blue staining showed that no cell deaths occurred when NCI-H441 cells were exposed to BrdU at concentrations of 100 μM or less (data not shown). However, when the cells were exposed to BrdU at 25, 50, and 100 μM for 10 days, they dose-dependently displayed senescence phenotypes, as exemplified by increased SA β-gal activity (Figure 5A), a distinct, flat, and enlarged morphology (Figure 5A), growth arrest (Figure 5B), and p21 expression (Figure 5C). When NCI-H441 cells were exposed to BrdU at any of these three concentrations for 10 days, washed in PBS, and then stimulated with 10% FCS for 3 days, cell growth did not resume, confirming the irreversibility of the senescence growth arrest (data not shown). In addition, the cellular senescence induced by BrdU exposure was accompanied by phosphorylation of H2AX (γH2AX) (Figure 5D), suggesting that the genotoxic stress imposed by BrdU contributed to the induction of senescence [15-18]. To investigate whether cell senescence impairs the self-repair capacity of epithelial cells, monolayers of NCI-H441cells cultured in the presence or absence of 25 μM BrdU were mechanically damaged. The damaged area in BrdU-exposed monolayers was repopulated more slowly than that in unexposed monolayers (Figure 5E), suggesting that cell senescence impaired epithelial wound repair.

**Figure 6:**
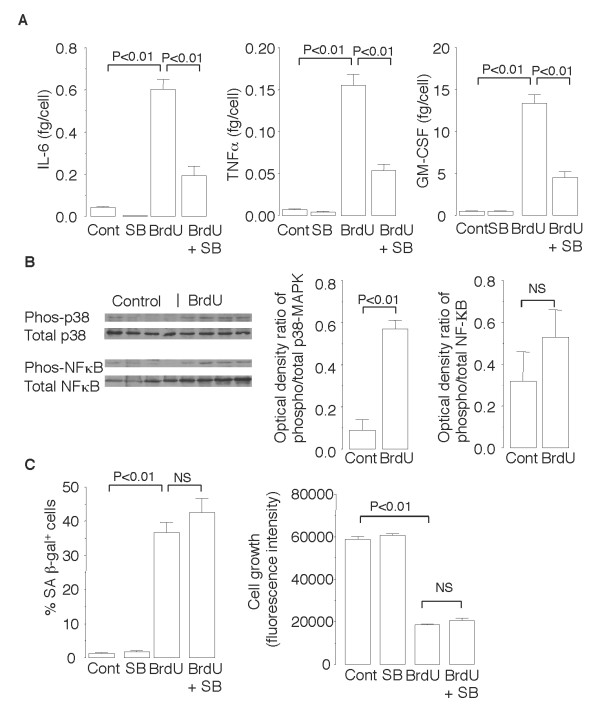
**Senescence of NCI-H441 cells after exposure to BrdU for 10 days is accompanied by p38 MAPK-dependent pro-inflammatory cytokine production**. (*A*) ELISA to measure concentrations of IL-6, TNF-α, and GM-CSF in the culture supernatants of NCI-H441 cells exposed or not exposed to 25 μM of BrdU in the presence or absence of 10 μM of the p38 MAPK inhibitor SB202190. The concentration of the anti-inflammatory cytokine IL-10 was below the limit of detection. (*B*) Immunoblot analyses for phosphorylation levels of p38 MAPK and NF-κB in the cell lysates. (*C*) Effects of SB202190 on BrdU-induced SA β-gal activation and growth arrest. Data are expressed as the means ± SEM. *N *= 3-6 in each experiment.

**Figure 7:**
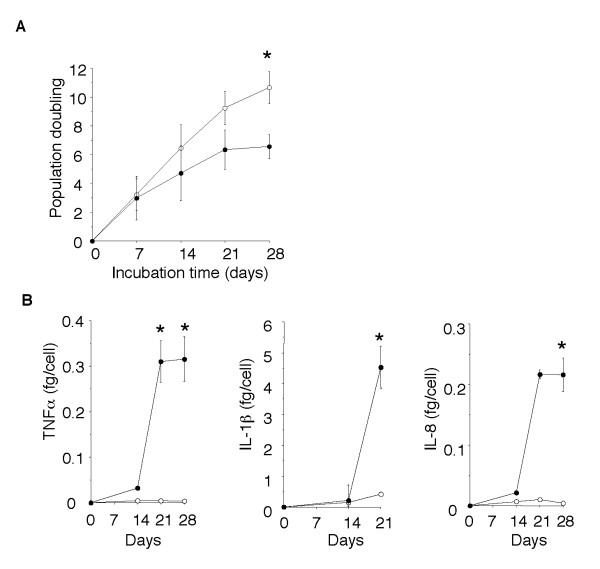
**The telomerase inhibitor MST-312 induces cellular senescence and pro-inflammatory cytokine production**. NCI-H441 cells were cultured for 28 days in the presence (*closed circles*) or absence (*open circles*) of 2.5 μM MST-312, with passages every 7 days. (*A*) Population doubling of NCI-H441 cells exposed and not exposed to the telomerase inhibitor. (*B*) Concentrations of pro-inflammatory cytokines in the culture supernatants. Data are expressed as the means ± SEM. *P < 0.05 and **P < 0.01 vs. cells not exposed to BrdU. *N *= 3-5 in each group.

As shown in Figure 6A, NCI-H441 cells exposed to BrdU for 10 days secreted 15- to 30-times greater amounts of the pro-inflammatory cytokines IL-6, TNFα, and GM-CSF than unexposed cells secreted. However, the amount of the anti-inflammatory cytokine IL-10 secreted by both the BrdU-exposed cells and unexposed cells was below the limit of detection (< 3.1 pg/ml), suggesting that a pro-inflammatory shift occurred after BrdU exposure. Exposure to BrdU for only 24 hours did not stimulate NCI-H441 cells to secrete pro-inflammatory cytokines (0.33 ± 0.02 fg/cell GM-CSF secreted by BrdU-exposed cells vs. 0.24 ± 0.07 fg/cell GM-CSF secreted by control cells, P = 0.38), indicating that the pro-inflammatory cytokine secretion in response to BrdU was not due to a direct stimulatory effect on the cells. To determine whether senescence inducers other than BrdU also increase pro-inflammatory cytokine secretion, NCI-H441 cells were cultured for 30 days in the presence or absence of the telomerase inhibitor MST-312 [20]. Exposure to MST-312 induced senescence growth arrest and markedly increased secretion of TNFα, IL-1β, and IL-8 by NCI-H441 cells (Figure 7). These results suggest that the increase in senescence-associated pro-inflammatory cytokine secretion was not an effect that was peculiar to BrdU.

The signaling pathways that lead to pro-inflammatory cytokine secretion usually involve activation of various molecules, including NF-κB and p38 MAPK. Immunoblot analyses showed that exposure of NCI-H441 cells to BrdU for 10 days significantly increased phosphorylation of p38 MAPK but not of NF-κB (Figure 6B). Furthermore, treatment of NCI-H441 cells with the p38 MAPK inhibitor SB202190 substantially reduced the increases in levels of IL-6, TNFα, and GM-CSF secreted by BrdU-exposed cells (Figure 6A). By contrast, SB202190 did not inhibit the BrdU-induced growth arrest or SA β-gal activation (Figure 6C). These results suggest that p38 MAPK activation is required for the senescence-associated pro-inflammatory cytokine secretion after induction of NCI-H441 cell senescence by BrdU but not for the growth arrest.

### P38 MAPK inhibitor suppresses senescence-associated inflammation in murine airways

**Figure 8:**
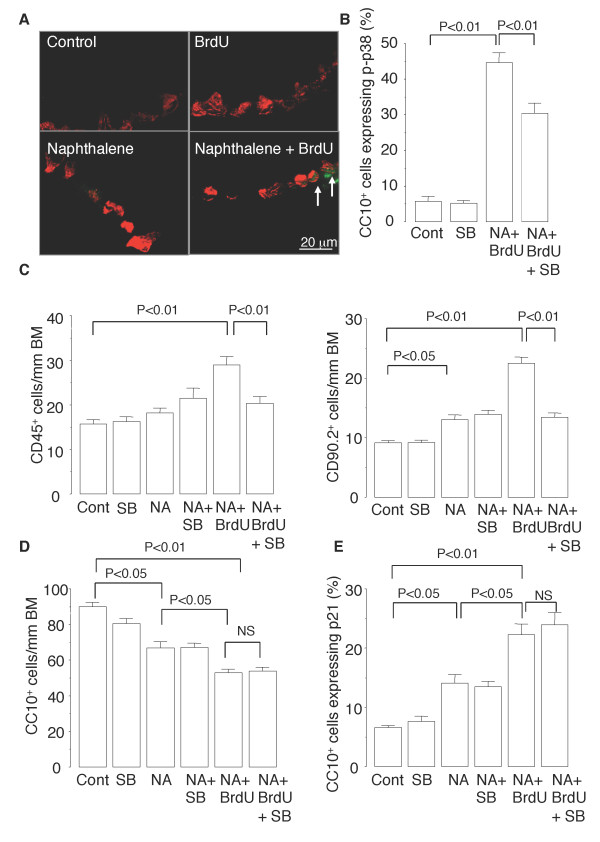
**P38 MAPK inhibitor suppresses senescence-associated airway inflammation in mice**. (*A*) Representative photomicrographs of double immunofluorescence staining for phospho-p38 MAPK (*green*) and CC10 (*red*) in the distal airway. *Arrows *indicate CC10-positive Clara cells that express phospho-p38 MAPK in their nuclei. Treatment with SB202190 of mice repeatedly exposed to NA and injected with BrdU reduces the proportion of Clara cells that express phospho-p38 MAPK (*B*) and the numbers of CD45-positive cells and CD90.2-positive cells infiltrating the distal airways (*C*) but does not affect the number of Clara cells (*D*) or the number of Clara cells that express p21 (*E*). Data are expressed as the means ± SEM. *N *= 4-6 in each experiment.

Next, we investigated whether SB202190 would inhibit senescence-associated inflammation in murine airways. The percentage of CC10-positive cells that expressed phospho-p38 MAPK was higher in the mice repeatedly exposed to NA and BrdU than in the control mice (Figure 8A and 8B). Treatment of the mice with SB202190 reduced not only the increase in the proportion of CC10-positive cells that expressed phospho-p38 MAPK (Figure 8B) but the increases in numbers of CD45-positive cells and CD90.2-positive cells that infiltrated the distal airways (Figure 8C). By contrast, SB202190 did not inhibit the reduction in the number of CC10-positive cells or the increase in the percentage of CC10-positive cells that expressed p21 in the distal airways of the mice (Figure 8D and 8E). These results suggest that SB202190 inhibits senescence-associated inflammation but not senescence growth arrest in the murine model of BrdU-induced epithelial senescence.

### P38 MAPK activation in senescent Clara cells in the airways of COPD patients

**Figure 9:**
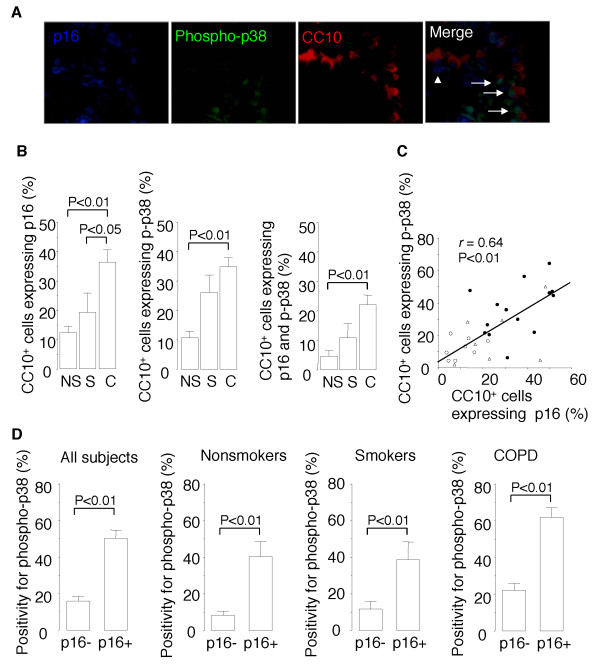
**P38 MAPK activation in senescent Clara cells in the airways of COPD patients**. (*A*) Representative photomicrographs of triple immunofluorescence staining of lung tissue sections obtained from COPD patients. *Arrows *indicate CC10-positive Clara cells that express both p16 and phospho-p38 MAPK. The *arrowhead *indicates a CC10-positive cell that expresses p16 but not phospho-p38 MAPK (*B*) Percentages of CC10-positive Clara cells that express p16, CC10-positive Clara cells that express phospho-p38 MAPK, and CC10-positive Clara cells that express both p16 and phospho-p38 MAPK in the lungs of COPD patients (*C*: *n *= 14), asymptomatic smokers (*S*: *n *= 7), and asymptomatic nonsmokers (*NS*: *n *= 8). (*C*) Correlation between the percentage of CC10-positive Clara cells that express p16 and the percentage of CC10-positive Clara cells that express phospho-p38 MAPK. *Open circles *= asymptomatic nonsmokers; *open triangles *= asymptomatic smokers; *closed circles *= COPD patients. (*D*) Rates of immunopositivity for phospho-p38 MAPK in CC10-positive Clara cells that express p16 (senescent Clara cells) and in CC10-positive Clara cells that do not express p16 (presenescent Clara cells) in the subjects as a whole, asymptomatic nonsmokers, asymptomatic smokers, and COPD patients.

The results obtained in the experiments on mice and cell cultures suggested that BrdU induces senescence of epithelial cells (Clara cells and NCI-H441 cells) that is accompanied not only by impaired epithelial regeneration but also by p38 MAPK-dependent exacerbation of the inflammatory response. We therefore investigated whether Clara cell senescence is accelerated in the airways of COPD patients, and if so, whether it is accompanied by p38 MAPK activation. The distal airway epithelium of COPD patients was found to contain significantly higher percentages of CC10-positive cells that were positive for p16, CC10-positive cells that were positive for phospho-p38 MAPK, and CC10-positive cells that were positive for both p16 and phospho-p38 MAPK than the distal airway epithelium of asymptomatic nonsmokers (Figure 9A and 9B). When all of the subjects were included in a correlation analysis, the percentage of p16-positive Clara cells was found to be correlated with the percentage of phospho-p38 MAPK-positive Clara cells (Figure 9C). These results suggest that the Clara cells in the airways of COPD patients senesce more rapidly and express higher levels of p38 MAPK activation. We also found that a higher percentage of CC10-positive cells that were positive for p16 (i.e., senescent Clara cells) expressed phospho-p38 MAPK than CC10-positive cells that were negative for p16 (i.e., presenescent Clara cells), indicating that MAPK activation is correlated with senescence at the cellular level in vivo (Figure 9D). Higher positive phospho-p38 MAPK rates among senescent Clara cells than among presenescent Clara cells were observed in all of the subjects as a whole and in each of the subgroups, i.e., the COPD patients, asymptomatic smokers, and nonsmokers (Figure 9D). These results suggest greater activation of p38 MAPK in senescent Clara cells than in presenescent cells in both the presence and absence of COPD.

## Discussion

The results of the present study demonstrated that BrdU-induced senescence of airway epithelial cells impairs epithelial regeneration and stimulates p38 MAPK-dependent inflammation after NA-induced Clara cell depletion in mice. To our knowledge, this is the first evidence indicating that epithelial cell senescence contributes to incomplete repair and excessive inflammation in the airways of mice. The results of the study also showed for the first time that Clara cell senescence is accelerated in COPD patients and is accompanied by p38 MAPK activation, suggesting that epithelial cell senescence may contribute to the excessive inflammation in the airways of COPD patients.

We used BrdU as an inducer of premature senescence to model airway epithelial senescence in mice and using BrdU offered several advantages in the present study. First, induction of senescence by exposure to BrdU has well been established as a model of premature senescence in various types of cells [16-19]. Second, since NA selectively injures Clara cells, using NA in combination with BrdU facilitated selective induction of senescence of the airway epithelial cells, and allowed only proliferative epithelial cells to incorporate BrdU into their DNA during the cell division that commenced to restore the NA-depleted pool of Clara cells. This is supported by our findings that while BrdU induced senescence in an in vitro culture of proliferating NCI-H441 cells, BrdU itself did not induce senescence of quiescent airway epithelial cells in mice that had not been exposed to NA. We therefore think that the senescent CC10-positive cells found in the mice exposed to NA and BrdU were mostly derived from Clara cells, which are the major progenitors of cells in the distal airways, but may have included a subpopulation of Clara cells, such as vCE cells or BASCs, that function as progenitors capable of renewing NA-injured airway epithelium [26]. Third, immunostaining for Ki-67 (proliferation marker) and SA β-gal (senescence marker) in combination with BrdU immunostaining made it possible to track the fate of the epithelial cells that had incorporated BrdU into their DNA. In fact, we found that the epithelial cells that had incorporated BrdU into their DNA became senescent and no longer proliferated. However, a limitation of our study stems from the fact that the BrdU taken up by the cells is phosphorylated to deoxynucleotide monophosphate by the salvage pathway enzyme thymidine kinase, whose levels may differ from cell to cell [28], and thus the repeated BrdU injection of mice may have selected for a subset of cells that had a lower level of the salvage enzyme and were no longer able to incorporate BrdU into their DNA. Such selection may have biased the results of our study. Another limitation of our study is the fact that we used BrdU, not cigarette smoke, to induce cell senescence, which may make it uncertain to translate the results of animal experiments to human COPD.

However, our murine model of Clara cell senescence provided clear evidence that senescence impairs regenerative response to airway injury. This finding is not surprising because senescent cells no longer proliferate in response to growth stimulation [6,7]. The impaired regenerative response in the present study was not due to a direct cytotoxic effect of BrdU, because BrdU did not cause any discernible epithelial damage, and it did not exacerbate the NA-induced epithelial damage in the airway of the mice (Figure 1). By contrast, BrdU imposed genotoxic stress, as demonstrated by the phosphorylation of ATM/ATR substrates and γH2AX (Figure 3), which triggers the DNA damage signaling pathway that causes p21-dependent cell cycle arrest, and eventually an irreversible senescence arrest [6,7,29].

Recent evidence suggests that airway epithelial cells, including Clara cells, play a pro-inflammatory role in the immune response through secretion of pro-inflammatory cytokines [30,31]. In the present study we found that Clara cell senescence was accompanied by exacerbation of airway inflammation that was at least in part attributable to increased pro-inflammatory cytokine secretion by senescent epithelial cells (Clara cells). These findings corroborate those of previous studies showing that other senescence inducers, including oncogene activation, DNA damage, and telomere shortening, stimulate pro-inflammatory cytokine secretion by cultured fibroblasts and endothelial cells, a phenomenon termed the "senescence-associated secretory phenotype (SASP)" [10,32-38]. Our study also showed that senescent-associated inflammation occurs in vitro as well as in vivo, and identified p38 MAPK activation as a positive regulator of the senescence-associated inflammation. P38 MAPK activation is a crucial step in the synthesis of several pro-inflammatory cytokines and recent evidence indicates a critical role of the p38 MAPK pathway in proinflammatory cytokine production by cells that have undergone oncogene- and environmental stress-induced senescence [39,40]. Similar to the findings in our own study, a previous study showed that inhibition of p38 MAPK by SB202190 reduced expression of IL-8 by fibroblasts after oncogene-induced senescence [33]. Other potential regulators of senescence-associated inflammation include the transcription factors NF-κB and C/EBPβ [10,41]. Although no significant NF-κB activation in the BrdU-induced senescent NCI-H441 cells was detected in this study, in a previous study we found that NF-κB was activated in response to telomerase-inhibitor-induced senescence of alveolar type II-like A549 cells [42]. Since telomerase has been shown to locate to mitochondria, where it decreases ROS production, inhibition of telomerase may have increased the formation of ROS, and that may in turn have activated NF-κB [43]. Thus, the mechanism of senescence-associated inflammation may differ according to the cell types and senescence inducer. Our findings also suggest that the pathways that regulate the senescence-associated inflammation may be distinct from the pathways that regulate the senescence growth arrest, because the p38 MAPK inhibitor SB202190 substantially diminished senescence-associated inflammation (Figures 6A and 8C) but did not inhibit BrdU-induced growth arrest, p21 expression, or the increased SA β-gal activity (Figures 6C, 8D and 8E).

The increased pro-inflammatory cytokine secretion by senescent epithelial cells (Clara cells) may not be the sole mechanism responsible for the exacerbated airway inflammation in our murine model of epithelial cell senescence. Previous studies have shown that CC10, the major Clara cell secretory protein (CCSP), exerts anti-inflammatory effects and can attenuate airway inflammation through inactivation of secretory phospholipase A2 or regulation of macrophage behavior [44,45]. Thus, the reduced CC-10 levels in the airway fluid resulting from ineffective restoration of Clara cells due to senescence growth arrest may also contribute to the mechanism of the increased airway inflammation.

Pro-inflammatory cytokine secretion is one of the complex features of the senescence-associated secretory phenotype, which include disruption of normal tissue structure, promotion of endothelial cell invasion, and stimulation of tumor cell growth and invasion [10,37,46]. Why do senescent cells mount a pro-inflammatory cytokine response? Recent evidence suggests at least two important roles of senescence-associated pro-inflammatory cytokine secretion [10,37,46]. First, pro-inflammatory cytokines such as IL-6 and IL-8 act in an autocrine feedback loop to reinforce the senescence growth arrest and thereby reduce the risk of oncogenic transformation in a cell-autonomous manner [33,46]. Second, the pro-inflammatory cytokines mobilize innate immune cells, such as natural killer cells, that clear senescent cells [47,48]. These roles suggest that senescence-associated inflammation is important, especially early after senescence induction, to ensure efficient growth arrest and eventually to stimulate the immune system to clear senescent cells [10]. However, senescent cells accumulate in the tissues with age and in the affected tissues of patients with age-related diseases such as atherosclerosis and COPD, probably because either immune clearance is less efficient and/or the rate at which senescent cells are produced outpaces the rate of clearance [2,6,9,10]. Consequently, the deleterious effects of cellular senescence, i.e., impaired tissue restoration and chronic inflammation, may become apparent with time and contribute to the pathogenesis of age-related diseases.

If that is true, does cellular senescence contribute to the onset and progression of COPD? Our findings show accelerated senescence of Clara cells in the airways of COPD patients, and they extend the findings in previous studies, including our own previous study, demonstrating that various types of cells, including alveolar type II cells, endothelial cells, fibroblasts, and peripheral blood lymphocytes, senesced more rapidly in COPD patients than in control subjects [2-5]. In the present study we also demonstrated an increase in the phosphorylated form of p38 MAPK in the Clara cells of COPD patients, corroborating a previous study showing increased numbers of phospho-p38 MAPK-positive macrophages and phospho-p38 MAPK-positive alveolar cells in the lungs of COPD patients [49]. Importantly, we found that p38 MAPK is preferentially activated by senescent Clara cells rather than by presenescent cells, indicating a correlation between p38 MAPK activation and senescence at the cellular level in vivo. There is evidence that p38 MAPK activation plays a role in recruiting CD8 T lymphocytes into the lungs of COPD patients, and a p38 MAPK inhibitor has been shown to be effective in suppressing inflammation in a model of smoking-induced COPD in mice [49,50]. In light of all of this evidence, senescence-associated p38 MAPK activation in Clara cells appears to contribute to the onset and progression of airway inflammation in COPD.

## Conclusions

**Figure 10:**
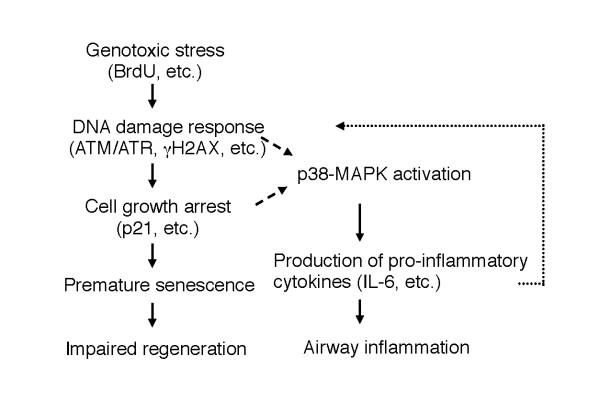
**Pathways by which BrdU impairs epithelial repair and induces persistent inflammation in the chronic NA injury model**. BrdU induces genotoxic stress, which activates the DNA damage response, thereby promoting premature senescence, which results in the growth arrest of epithelial cells. Genotoxic stress caused by BrdU also activates p38-MAPK pathways that trigger the production of pro-inflammatory cytokines/chemokines, which exacerbate inflammation. Which is necessary for p38-MAPK activation, the DNA damage response or cell cycle arrest (p21, etc.), has not been determined (*broken arrows*). Recent evidence indicates that pro-inflammatory cytokines (e.g., IL-6, IL-8) at least in part reinforce cell cycle arrest via the DNA damage response pathway [32,33], suggesting a positive feedback loop (*dashed arrow*) in which inflammation in turn promotes senescence.

The results of our study provide evidence that senescence of airway epithelial cells impairs repair processes and stimulates p38 MAPK-dependent inflammation in response to airway injury (Figure 10). Our findings are of clinical importance, because COPD is characterized by impaired repair and excessive inflammation and is associated with accelerated senescence of lung cells [9,51]. However, the results of study leave many questions unanswered. Is a DNA damage response or senescence growth arrest required for BrdU-induced senescence-associated inflammation to occur (Figure 10)? Do other senescence inducers, such as oxidative stress and cigarette smoke, also induce senescence-associated airway inflammation [21]? How long do senescent cells survive in the airway epithelium of mice and humans? Does senescence-associated inflammation account for the persistent airway inflammation in COPD patients who quit smoking? All of these questions need to be answered in the future.

## Abbreviations

COPD: chronic obstructive pulmonary disease; NA: naphthalene; BrdU: 5-bromo-2'-deoxyuridine; p38 MAPK: p38 mitogen-activated protein kinase; CYP: cytochrome P450; NBF: neutral buffered formalin; PD: population doubling; ELISA: enzyme-linked immunosorbent assay; SA β-gal: senescence-associated β-galactosidase; X-gal: 5-bromo-4-chloro-3-indoyl β-D galactoside; CC10: Clara cell 10-kDa secretory protein; p16: p16^INK4a^; p21: p21^WAF1/CIP1^; ATM/ATR: ataxia teleangiectasia mutated kinase/ataxia teleangiectasia and Rad3-related kinase; HRP: horseradish peroxidase; BM: basement membrane; ANOVA: analysis of variance; PI3K: phosphoinositide 3-kinase; SASP: senescence-associated secretory phenotype; CCSP: Clara cell secretory protein

## Competing interests

The authors declare that they have no competing interests.

## Authors' contributions

FZ carried out the animal studies, the cell culture studies, and the human lung tissue studies, and drafted the manuscript. SO carried out the human lung tissue studies. NA participated in the design of the study. KA conceived of the study, and participated in its design and coordination and helped to draft the manuscript. All authors read and approved the final manuscript.

## Supplementary Material

Additional file 1 **Additional methods**. The file contains detailed methods for epithelial repair assay, senescence-associated β-galactosidase staining, immunohistochemistry and immunofluorescence, and immunoblot analysis used in this study. Click here for file
