# Supplementary material for: Epithelial cell senescence impairs repair process and exacerbates inflammation after airway injury
Source: Respir Res. 2011 Jun 10;12(1):78. doi: 10.1186/1465-9921-12-78 (PMC3118351; doi:10.1186/1465-9921-12-78)
Supplement: Additional file 1 — Additional methods. The file contains detailed methods for epithelial repair assay, senescence-associated β-galactosidase staining, immunohistochemistry and immunofluorescence, and immunoblot analysis used in this study. [file 1465-9921-12-78-S1.PDF]

Additional File 1

Additional methods

Epithelial Cell Senescence Impairs Repair Process and Exacerbates Inflammation After Airway Injury

Fang Zhou, Shigemitsu Onizawa, Atsushi Nagai, and Kazutetsu Aoshiba

METHODS

Epithelial repair assay

NCI-H441 cells were grown on 30 mm-plates and cultured in RPMI 1640 supplemented with 10% FCS in the presence or absence of 25 μM BrdU for 10 days. Cell monolayers were then damaged mechanically by crossing three times with a 10–200 μL volume universal pipette tip (Corning, NY, USA). After washing twice with phosphate-buffered saline cells were allowed to repopulate the damaged area. Immediately after mechanical damage (t = 0) and at 24, 48, and 72 hours cell monolayers were observed under a phase contrast microscope (Olympus BX60: Olympus Optical Co., Ltd., Tokyo, Japan), photographed, and the area of unpopulated cells was calculated with image analysis (Win Roof Version 3.5; Mitani Corporation, Fukui, Japan).

Senescence-associated β-galactosidase (SA β-gal) staining

SA β-gal staining was performed as described previously [1]. Cell monolayers or frozen tissue sections were fixed with 2% formaldehyde and 0.2% glutaraldehyde in PBS for 5 minutes at room temperature. The slides were then rinsed with PBS and incubated with a SA β-gal staining solution containing 40 mM sodium citrate (pH 6.0), 150 mM NaCl, 5 mM potassium ferricyanide, 5 mM potassium ferrocyanide, 2 mM $MgCl_2$, and 1 mg/ml of 5-bromo-4-chloro-3-indoyl β-D galactoside (X-gal, Sigma).

Immunohistochemistry and Immunofluorescence

The primary antibodies used were goat polyclonal anti-Clara cell 10-kDa secretory protein (CC10) (Santa Cruz Biotechnology, Inc., Santa Cruz, CA), mouse monoclonal anti-β-tubulin IV (BioGenex, San Ramon, CA), rabbit polyclonal anti-Ki-67 (Abcam,

Tokyo, Japan), mouse monoclonal anti-BrdU (Chemicon, Temecula, CA), mouse monoclonal anti-p16^{INK4a} (p16) (Santa Cruz), rabbit polyclonal anti- p21$^{WAF1/CIP1}$ (p21) (Abcam), rabbit monoclonal anti-phospho(Thr180/Tyr182)-p38 MAPK (Cell Signaling Technologies, Danvers, MA), rabbit polyclonal anti-phospho(Ser/Thr)-ataxia teleangiectasia mutated kinase (ATM)/ataxia teleangiectasia and Rad3-related kinase (ATR) substrate, rabbit monoclonal anti-phospho(Ser139)-H2AX (γH2AX, Cell Signaling), rat monoclonal anti-CD45 (BD Pharmingen, Tokyo, Japan), and rat monoclonal anti-CD90.2 (BD Pharmingen). For immunohistochemistry and immunocytochemistry, the primary antibodies were detected with a secondary antibody conjugated with a horseradish-peroxidase-labeled polymer (Envison+®, DAKO Japan, Tokyo, Japan; Histofine® Simple Stain, Nichirei Biosciences, Tokyo Japan). To expose the immunoreactive epitopes of antigens in paraffin-embedded tissue samples, the sections were autoclaved in a citrate buffer (10 mM, pH 6.0) for 30 minutes before application of the primary antibodies. Endogenous peroxidase activity was quenched by exposure to 3% peroxide for 20 minutes. Immunoreactants were detected with a diaminobenzidine substrate. For double staining, the prior antibody complexes were erased by immersing the slides in glycine-HCl buffer (pH2.2) for one hour, and the sections were then immunostained for the secondary antigen. Immunoreactants against the secondary antigen were detected by using a HistoGreen® substrate detection kit for peroxidase (AbCys, Paris, France). For immunofluorescence staining, the primary antibodies were reacted with secondary anti-IgG antibodies conjugated with Alexa Fluor 350, Alexa Fluor 488, or Alexa Fluor 594 (Invitrogen, Carlsbad, CA). Images

were acquired by using an Olympus BX60 microscope (Olympus Optical Co., Ltd., Tokyo, Japan) equipped with a digital camera, and processed with a computerized color image analysis software system (Win Roof Version 3.5; Mitani Corporation, Fukui, Japan) and Adobe Photoshop software (San Jose, CA). The numbers of γH2AX-foci in the cell nuclei of at least 50 cells were counted visually through an Olympus BX60 microscope equipped with a 100x objective as described previously [2, 3].

Immunoblot Analysis

Cell lysates were solubilized in RIPA buffer (0.15 M NaCl, 50 mM Tris-Cl, pH 7.4, 0.5% NP40, and 0.1% sodium dodecyl sulfate) containing 10 μg/ml leupeptin, 1 mM PMSF, 10 μg/ml aprotinin, and 1 mM sodium vanadate. Samples were then fractionated by sodium dodecyl sulfate-polyacrylamide gel electrophoresis and transferred to a polyvinylidene difluoride membrane. The membrane was probed with rabbit monoclonal anti-phospho(Thr180/Tyr182)-p38 MAPK, rabbit monoclonal anti-p38 MAPK, rabbit polyclonal anti-NF-κB p65 (Cell Signaling), rabbit polyclonal anti-phospho-NF-κB p65 (Ser536) (Cell Signaling), rabbit monoclonal anti-phospho(Ser139)-H2AX (γH2AX, Cell Signaling), rabbit polyclonal anti-p21, or rabbit polyclonal anti-actin (Sigma). The primary antibodies were detected with an HRP-conjugated antibody, which in turn was visualized by enhanced chemiluminescence (SuperSignal® West Femto; Pierce, Rockford, IL). Signal intensity was analyzed with the free NIH image PC software.

References

1. Tsuji T, Aoshiba K, Nagai A: **Cigarette smoke induces senescence in alveolar epithelial cells.** *Am J Respir Cell Mol Biol* 2004, **31**:643-649.

2. Mah LJ, EL-Osta A, Karagiannis TC: **γH2AX: a sensitive molecular marker of DNA damage and repair.** *Leukemia* 2010, **24**:679-686.

3. Rübe CE, Fricke A, Wendorf J, Stützel A, Kühne M, Ong MF, Lipp P, Rübe C: **Accumulation of DNA double-strand breaks in normal tissues after fractionated irradiation.** *Int J Radiation Oncol Biol Phys* 201, **76**:1206-1213.
